# Pain Management and Opioid Use with Long-Acting Peripheral Nerve Blocks for Hand Surgery: A Descriptive Study

**DOI:** 10.5812/aapm-139454

**Published:** 2023-10-29

**Authors:** Brandon W Knopp, Emma Eng, Ehsan Esmaeili

**Affiliations:** 1 Medical Student, Department of Anesthesiology, Charles E. Schmidt College of Medicine at Florida Atlantic University, Boca Raton, Florida, United States; 2 Department of Anesthesiology, Charles E. Schmidt College of Medicine, Florida Atlantic University, Boca Raton, Florida, United States; 3 Department of Orthopedic Surgery, Charles E. Schmidt College of Medicine, Florida Atlantic University, Boca Raton, Florida, United States

**Keywords:** Outpatient Surgery, Nerve Block, Analgesics, Opioid, Pain Management, Bupivacaine, Anesthesiology, Pain, Postoperative

## Abstract

**Background:**

Peripheral nerve blocks (PNBs) are used in multiple surgical fields to provide a high level of regional pain relief with a favorable adverse effect profile. Peripheral nerve blocks aim to decrease overall perioperative pain and lower systemic analgesic requirements. Short-acting anesthetic agents are commonly given as single-injection PNBs for pain relief, typically lasting less than 24 hours. Liposomal bupivacaine is a newer anesthetic formulation lasting up to 72 hours as a single-injection PNB and may allow patients to recover postoperatively with a lower need for opioid analgesics.

**Objectives:**

This study investigates peri- and postoperative pain and opioid use in patients receiving a long-acting brachial plexus PNB for hand surgery.

**Methods:**

A retrospective review of patients who underwent a long-acting PNB using liposomal bupivacaine in the brachial plexus for minor hand operations was performed between July 2020 and May 2023 in Florida, USA. Patients were administered a ten-question survey regarding perioperative pain levels, post-operative symptoms, patient satisfaction, postoperative opioid use, and postoperative non-opioid analgesics.

**Results:**

One hundred three patients, including 21 males and 82 females with an average age of 68.3 ± 15.8 years, completed a survey (34.2% response rate). Patients reported a considerable reduction in pain from 7.9 ± 2.2 out of ten before the PNB to 1.6 ± 1.8 in the perioperative period, 4.3 ± 2.7 in postoperative days zero to three, and 3.8 ± 2.4 in postoperative days four and five. Nerve block effects lasted a mean of 2.2 ± 2.0 days and patients reported a high level of satisfaction regarding their pain management plan with a score of 9.4 ± 1.4 out of ten. 20.4% of patients were prescribed opioids and 41.7% used NSAIDs postoperatively.

**Conclusions:**

Liposomal bupivacaine PNBs effectively reduced peri- and postoperative pain with pain relief lasting 2.2 ± 2.0 days. Patients were highly satisfied with their pain management and there was a low rate of postoperative opioid prescription. Given these results, long-acting PNBs have the potential to significantly improve patient satisfaction, reduce anesthesia use, and reduce postoperative opioid prescription.

## 1. Background

Peripheral nerve blocks (PNBs) are increasingly used in multiple surgical subspecialties including orthopedic surgery, plastic surgery, abdominal surgery, and other surgical fields. Peripheral nerve blocks are on the fourth step of the modified WHO analgesic ladder and provide a high level of regional pain relief with a favorable adverse effect profile (1). These injections can offer more effective regional pain relief than general anesthesia or oral pain medications and isolate the areas requiring pain control (2, 3). They are particularly useful in surgeries involving the extremities or regions innervated by one primary nerve plexus, such as a celiac plexus block for pancreatic cancer resection (4). This study investigates a long-acting PNB and reports peri- and postoperative pain experienced by patients undergoing outpatient hand surgeries following a brachial plexus block.

Patient satisfaction following PNBs is high according to several studies that reported improved pain control compared to opioids in the first 24 hours after several common surgical procedures (3, 5, 6). By providing adequate pain control for select operations, PNBs can reduce the need for general anesthesia and postoperative opioid pain control. In the operative and immediate postoperative periods, PNBs are associated with a decreased need for rescue analgesics for breakthrough pain (7). Peripheral nerve blocks may be preferable in patients at high risk of respiratory depression and other adverse events from general anesthesia, patients unable to tolerate oral medications, patients where avoidance of opioid use is preferable, and in procedures with a high degree of pain where an augmentation of general anesthesia and postoperative pain control is needed (8). Peripheral nerve blocks are typically well-tolerated as well. Adverse events are rare but may include infection, bleeding, allergic reactions, damage to surrounding structures, nerve injury, and systemic toxicity due to intravascular uptake of local anesthetic (9). There are few contraindications to PNBs, including local infection at the injection site, body habitus preventing ultrasonographic visualization, coagulopathy, blood thinner use, pre-existing neurological deficit, and inability to cooperate with placement (2, 9).

Peripheral nerve blocks can be administered as a single injection or a continuous nerve block via a percutaneous catheter. Each modality presents similar risks of adverse events, though a single injection is more convenient for outpatient procedures not requiring extended observation or inpatient admission (10, 11). Short-acting agents such as lidocaine, mepivacaine, ropivacaine, and bupivacaine are commonly used in single-injection PNBs to provide perioperative anesthesia and analgesia typically lasting less than 24 hours (12). However, longer-acting formulations such as liposomal bupivacaine are gaining attention for peri- and postoperative pain relief which may last up to 72 hours. Liposomal bupivacaine is Food and Drug Administration-approved for transversus abdominis plane and interscalene nerve blocks for postoperative pain relief following shoulder surgery. However, additional uses for this long-acting regional anesthetic formulation are being investigated. Long-acting regional anesthesia via a single injection is beneficial as it may decrease the need for opioids in the first several postoperative days and reduce the potential for opioid misuse and overprescription.

The link between postoperative opioid prescription and opioid misuse has been widely recognized, as opioids are often ineffective at properly addressing moderate to extreme postoperative pain. Patients only take 34% of their prescribed opioid pills postoperatively, according to Hill et al. 2017, potentially indicating an overprescription of opioids (13). Alternatively, the underuse of prescribed opioid pills postoperatively may be due to inadequate regional pain relief or intolerable adverse effects. However, there is still a considerable risk of opioid misuse in patients who did not complete their postoperative opioid prescription (14). One study found that about 6.0% of opioid-free adults in the year before surgery used opioids beyond postoperative day 90 (15). As long-acting PNBs can offer postoperative pain relief for up to 72 hours after surgery, they have been investigated as a means of reducing opioid prescription. A 2022 study by Kiefhaber and Vyrva found reduced opioid use in the first five days after surgery following regional liposomal bupivacaine injection but no differences in postoperative pain scores (16). While some evidence suggests liposomal bupivacaine PNBs are useful in select procedures, there is scant research regarding the use of these injections in hand surgeries.

## 2. Objectives

This study investigates the subjective peri- and postoperative pain experienced by patients undergoing hand surgery and postoperative opioid use following a long-acting brachial plexus block using liposomal bupivacaine. The authors postulate that these long-acting PNBs will provide adequate peri- and postoperative anesthesia while reducing the need for opioid prescription.

## 3. Methods

A retrospective review of patients who underwent a long-acting PNB for minor hand operations between July 2020 and May 2023 in Florida, USA was performed. The PNBs were placed in the brachial plexus using liposomal bupivacaine as the anesthetic agent. Hand surgeries included basal thumb joint reconstruction, carpal tunnel release, digital arthroplasty, extensor indicis proprius (EIP) to extensor pollicis longus (EPL) tendon transfer, digital arthroplasty, digital fracture reduction, and open reduction of the wrist or distal radius. Surgeries were done in an ambulatory surgical center using a liposomal bupivacaine anesthetic agent and general anesthesia as indicated. All patients had standard postoperative care by the anesthesiologist who placed the block, the nursing staff, and the operating surgeon. All patients had postoperative follow-up(s) per standard practice of the operating surgeon. Peripheral nerve blocks were placed while using ultrasonographic guidance for increased accuracy of the injection. A query identified patients who underwent minor hand operations utilizing long-acting PNBs. Inclusion criteria were: Age greater than 18, hand surgeries done in an ambulatory surgical center, and brachial plexus nerve blocks using liposomal bupivacaine. There were no exclusion criteria. Every patient was attempted to be contacted twice to administer a survey consisting of a ten-point Likert scale of agreement questions, numerical questions, and questions regarding adverse effects. Questions investigated pain severity in the pre-, peri- and postoperative periods, overall patient satisfaction, adverse effects, post-operative symptoms, additional analgesics used, and duration of nerve block effect (Appendix 1).

Patients were advised on non-pharmaceutical analgesic options and pharmaceutical analgesic options. The operating surgeon's standard practice is to recommend using non-opioid analgesics in patients who received a long-acting nerve block. Opioids were only prescribed as needed for breakthrough pain refractory to non-opioid agents. Patient demographic data, medical history, contact information, and surgical records were obtained via electronic health records (EHRs). Trained researchers administered surveys via telephone interview. Participants were informed of the study objective and gave informed consent to be included in the study before completing the telephone survey. Patients who could not be reached following the first call were called one additional time on a different day. Patients who declined to participate or did not answer either phone call were excluded from the study.

### 3.1. Ethical Statements

The study was performed in accordance with the ethical standards established by the Declaration of Helsinki (1964) and all subsequent amendments. This study is reported using the Strengthening the Reporting of Observational Studies in Epidemiology (STROBE) guidelines.

## 4. Results

One hundred three patients met inclusion criteria, consented to be included in the study, and completed a survey (34.2% response rate). The study group included 21 male and 82 female patients with an average age of 68.3 ± 15.8 years. Thirty-three patients reported current or former tobacco use, though the medium and amount of tobacco use were not reported. The surgeries reported in this study are shown in Table 1. Questions 1 - 5 described overall patient satisfaction with pain management and pre-, peri- and postoperative pain (Table 2). Survey responses showed a low level of perioperative pain and a considerable reduction in pain from the preoperative period to the first five days following surgery. Nerve block effects lasted a mean of 2.2 ± 2.0 days, ranging from several hours to 14 days.

**Table 1.** Surgeries Reported Using Long-Acting PNB

Surgeries	Number of Patients
**Basal joint reconstruction**	17
**Open reduction of the wrist or distal radius**	66
**Carpal tunnel release**	7
**Digital fracture reduction**	6
**Digital arthroplasty**	3
**EIP to EPL tendon transfer**	4

Abbreviations: PNB, peripheral nerve block; EIP, extensor indicis proprius; EPL, extensor pollicis longus.

**Table 2.** Perioperative Pain Scores

Variables	Likert Score, Mean ± SD
**Preoperative pain level**	7.9 ± 2.2
**Perioperative pain**	1.6 ± 1.8
**Pain during postoperative days 0 - 3**	4.3 ± 2.7
**Pain during postoperative days 4 - 5**	3.8 ± 2.4
**Overall satisfaction with pain management**	9.4 ± 1.4

Patients reported high satisfaction with their pain management plan, with a mean response of 9.4 ± 1.4 out of ten. Six patients (5.8%) reported pain or swelling around the injection site and none required follow-up care for injection site-related complications. Seven patients (6.8%) reported adverse effects following surgery after receiving a liposomal bupivacaine PNB. Four patients (3.9%) reported nausea, two patients (1.9%) reported lightheadedness, and one patient (1.0%) reported vertigo after injection. No patients experienced motor dysfunction or allodynia in the peri- or postoperative periods. Twenty-one patients (20.4%) reported opioid use postoperatively. Forty-three patients (41.7%) used NSAIDs independently or in addition to opioids following surgery.

## 5. Discussion

Liposomal bupivacaine is a long-acting anesthetic agent increasingly used in PNBs. This formulation has been reported to relieve regional pain for up to 72 hours postoperatively, compared to other regional anesthetics which last around six to 24 hours after injection, depending on formulation and location (17, 18). The Food and Drug Administration has approved liposomal bupivacaine for transversus abdominis plane and interscalene nerve blocks, but additional uses are being investigated (19, 20). This study reports on brachial plexus blocks utilizing liposomal bupivacaine for minor hand surgeries performed in an outpatient setting.

The results show that liposomal bupivacaine nerve blocks effectively reduce peri- and postoperative pain. Patients reported significant preoperative pain at 7.9 ± 2.2 out of ten. Perioperative pain, by comparison, was well-controlled with a combination of monitored anesthesia care (MAC) and the PNB as patients reported a mean score of 1.6 ± 1.8 out of ten. Postoperative pain in postoperative days zero to three and days four and five were reported as 4.3 ± 2.7 out of ten and 3.8 ± 2.4 out of ten, respectively. This mild pain level shows the potential for liposomal bupivacaine to bridge patients through the first postoperative days with a lower additional pain relief requirement.

Overall, patients reported high satisfaction with their pain management plan at 9.4 ± 1.4 out of ten and only three patients (2.9%) described the postoperative period as “painful”. PNB injections are associated with a low risk of adverse events as only seven patients (6.8%) reported minor adverse effects including nausea, lightheadedness, and vertigo. While some adverse effects may have been due to the MAC, this low level of minor adverse events should not limit the use of liposomal bupivacaine as a PNB agent. The 20.4% of patients prescribed opioids in this study is a significant reduction compared to the 59% to 76% of hand surgery patients prescribed opioids postoperatively (21-23). Adequate postoperative pain control via long-acting PNBs allows reduced opioid prescription as the greatest consumption of opioids occurs on postoperative days zero and one, with median days of total consumption between two to five days (21, 24). Additional pain relief may be required for patients with breakthrough pain or in whom the long-acting PNB effects wane early, however, for most patients this pain control method is adequate in the early postoperative period.

Reducing opioid prescription following hand surgery is a key benefit of the long-acting liposomal bupivacaine PNBs. Studies have shown that even though postoperative pain is frequently not treated effectively, only around $\frac{1}{3}$ of prescribed opioids are used (24). This underuse of prescribed opioids not only highlights the inadequacy of opioids for postoperative pain, but also contributes to the potential misuse of leftover tablets by the patient or a third party (25). According to the results of this study, long-acting PNBs may be an effective method of improving pain management and decreasing reliance on prescribing opioids for postoperative pain from minor hand surgeries.

The results of this study suggest that long-acting PNBs can provide adequate pain management and reduce opioid prescriptions following several minor hand surgeries. However, further research should aim to prospectively investigate this potential association. This study is limited by the method of surveying patients via the phone, however, reported opioid use and pain scores were confirmed during standard postoperative follow-up visits. Despite the limitations of this study, the results justify further investigation into the use of long-acting PNBs for hand surgeries and other operations. By providing effective, extended pain management postoperatively, long-acting PNBs have the potential to significantly improve patient satisfaction, reduce general anesthesia use, and reduce postoperative opioid prescription.

aapm-13-5-139454-s001.docx

## Data Availability

The dataset presented in the study is available on request from the corresponding author during submission or after publication. The data are not publicly available due to patient privacy reasons.
