# Supplementary figures and images for: Pain Management and Opioid Use with Long-Acting Peripheral Nerve Blocks for Hand Surgery: A Descriptive Study
Source: Anesth Pain Med. 2023 Oct 29;13(5):e139454. doi: 10.5812/aapm-139454 (PMC10998466; doi:10.5812/aapm-139454)

**Appendix 1: Patient Survey Questions**


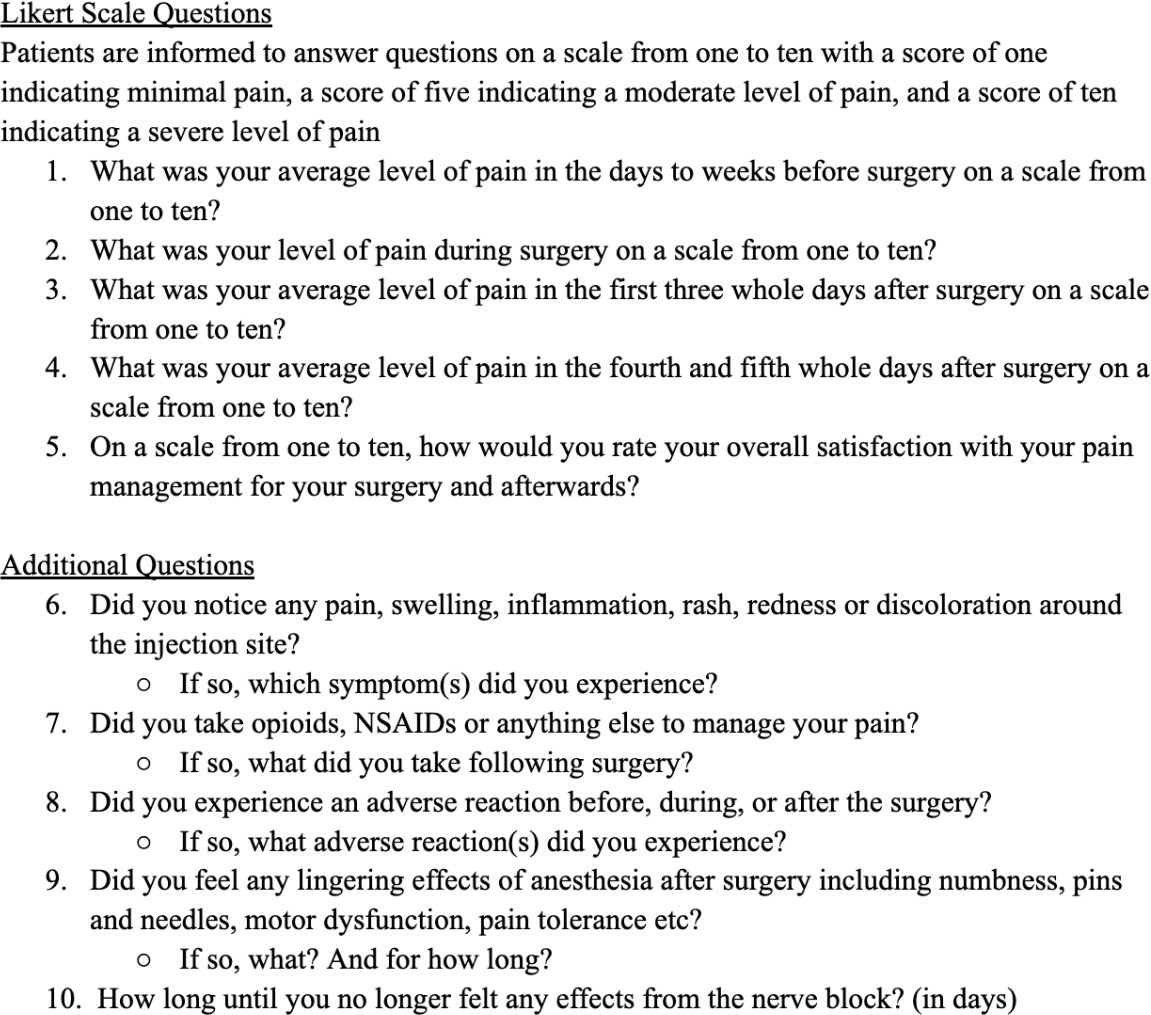

Supplement: aapm-13-5-139454-s001.docx [file aapm-13-5-139454-s001.docx]
